# 4-phenylbutyrate Mitigates Fluoride-Induced Cytotoxicity in ALC Cells

**DOI:** 10.3389/fphys.2017.00302

**Published:** 2017-05-11

**Authors:** Maiko Suzuki, Eric T. Everett, Gary M. Whitford, John D. Bartlett

**Affiliations:** ^1^Division of Biosciences, College of Dentistry, The Ohio State UniversityColumbus, OH, USA; ^2^Department of Pediatric Dentistry and The Carolina Center for Genome Sciences, University of North CarolinaChapel Hill, NC, USA; ^3^Department of Oral Biology, College of Dental Medicine, Georgia Regents UniversityAugusta, GA, USA

**Keywords:** fluoride, dental fluorosis, enamel, ameloblast, 4-phenylbutyrate, ER stress, apoptosis, TGF-β1

## Abstract

Chronic fluoride over-exposure during pre-eruptive enamel development can cause dental fluorosis. Severe dental fluorosis is characterized by porous, soft enamel that is vulnerable to erosion and decay. The prevalence of dental fluorosis among the population in the USA, India and China is increasing. Other than avoiding excessive intake, treatments to prevent dental fluorosis remain unknown. We previously reported that high-dose fluoride induces endoplasmic reticulum (ER) stress and oxidative stress in ameloblasts. Cell stress induces gene repression, mitochondrial damage and apoptosis. An aromatic fatty acid, 4-phenylbutyrate (4PBA) is a chemical chaperone that interacts with misfolded proteins to prevent ER stress. We hypothesized that 4PBA ameliorates fluoride-induced ER stress in ameloblasts. To determine whether 4PBA protects ameloblasts from fluoride toxicity, we analyzed gene expression of *Tgf*-β*1, Bcl2*/*Bax* ratio and cytochrome-c release *in vitro*. *In vivo*, we measured fluorosis levels, enamel hardness and fluoride concentration. Fluoride treated Ameloblast-lineage cells (ALC) had decreased *Tgf*-β*1* expression and this was reversed by 4PBA treatment. The anti-apoptotic *Blc2*/*Bax* ratio was significantly increased in ALC cells treated with fluoride/4PBA compared to fluoride treatment alone. Fluoride treatment induced cytochrome-c release from mitochondria into the cytosol and this was inhibited by 4PBA treatment. These results suggest that 4PBA mitigates fluoride-induced gene suppression, apoptosis and mitochondrial damage *in vitro*. *In vivo*, C57BL/6J mice were provided fluoridated water for six weeks with either fluoride free control-chow or 4PBA-containing chow (7 g/kg 4PBA). With few exceptions, enamel microhardness, fluorosis levels, and fluoride concentrations of bone and urine did not differ significantly between fluoride treated animals fed with control-chow or 4PBA-chow. Although 4PBA mitigated high-dose fluoride toxicity *in vitro*, a diet rich in 4PBA did not attenuate dental fluorosis in rodents. Perhaps, not enough intact 4PBA reaches the rodent ameloblasts necessary to reverse the effects of fluoride toxicity. Further studies will be required to optimize protocols for 4PBA administration *in vivo* in order to evaluate the effect of 4PBA on dental fluorosis.

## Introduction

Dental caries remains the most common chronic disease in which acid produced by bacteria dissolves tooth enamel (Dye et al., 2017). Dental caries is a largely preventable condition and fluoride has proven an effective caries prophylactic. The U.S. Public Health Service (PHS) recommends public water fluoridation at an optimal fluoride concentration of 0.7 ppm (corresponding to 0.04 mM NaF) in order to prevent dental caries (Health and Human Services Federal Panel on Community Water, 2015). On the other hand, fluoride is an environmental health hazard and acute or chronic over-exposure can result in enamel fluorosis (Denbesten, 1999), skeletal fluorosis (Boivin et al., 1989), and reproductive toxicity in animal models (Sm and Mahaboob Basha, 2017).

The prevalence of dental fluorosis among the population in the USA, India, and China is increasing. Predominantly mild dental fluorosis among children aged 12–15 in USA is about 41% and represents an increase compared to the 1980s when it was 23% (Beltrán-Aguilar et al., 2010).

However, other than avoiding excessive intake during enamel development, treatments to prevent dental fluorosis remain unknown. Fluoride exerts diverse cellular effects in a dose, cell type, and tissue dependent manner. We and others have shown in several rodent tissues, including the enamel organ, that high-dose fluoride causes cell stress, such as endoplasmic reticulum (ER) stress (Kubota et al., 2005; Sharma et al., 2008; Ito et al., 2009) and oxidative stress (Sun et al., 2011; Suzuki et al., 2014b, 2015).

Enamel development occurs in stages, as defined by the morphology of the ameloblasts responsible for enamel formation. Secretory-stage ameloblasts secrete matrix metalloproteinase 20 (MMP20) and enamel proteins that combine to form a mineralization front that promotes appositional growth until the enamel layer reaches full thickness (Simmer et al., 2012; Bartlett and Smith, 2013). Maturation-stage ameloblasts secrete kallikrein related peptidase 4 (KLK4), reabsorb protein degradation products, and promote mass mineral deposition as the enamel hardens into its final form (Hu and Simmer, 2007). During the maturation stage ameloblasts are in direct contact with the acidic (pH <6.0) mineralizing enamel matrix (Smith et al., 1996). Therefore, maturation stage ameloblasts are exposed to fluoride under low pH conditions. The low extracellular pH surrounding the maturation stage ameloblasts promotes the conversion of F^−^ to HF. When the pKa value for HF (3.45) is substituted in the Henderson-Hasselbalch equation (pH = pKa+log [F^−^]/[HF]), we observe that at pH 7.4, the [F^−^]: [HF] ratio is 8913:1. However, at pH 6.0, this ratio decreases to 355:1. Therefore, approximately 25-fold more HF is formed at pH 6.0 as compared to pH 7.4. The low pH environment of maturation stage facilitates entry of toxic HF into ameloblasts to enhance fluoride-induced cell stress (Sharma et al., 2010). This suggests that compared to the secretory stage (pH ~ 7.2), the low pH environment of the maturation stage reduces the threshold dose required to induce fluoride-mediated cytotoxicity *in vivo*. In contrast, the *in vitro* cell culture environment (culture media) is neutral (pH ~ 7.3) which requires a higher fluoride dose than does a low pH environment to induce fluoride-mediated cytotoxicity. This suggests that the neutral cell culture environment *in vitro* requires a higher dose of fluoride than is present in serum to induce fluoride toxicity *in vitro*. It has previously been demonstrated that 100 fold disparity exists between fluoride sensitivity *in vitro* and *in vivo* (Bronckers et al., 2009).

The ER functions as a quality control organelle and prevents misfolded proteins from traversing the secretory pathway (Zhang et al., 1997). ER stress is caused by the accumulation of unfolded proteins. The response to this stress is known as the unfolded protein response (UPR) (Doyle et al., 2011). UPR activation results in transient suppression of protein translation, enabling cells to cope with the existing misfolded protein load. The UPR increases ER chaperone gene expression including GRP78/Bip to augment the folding capacity of the ER (Claudio et al., 2013).

Accumulated proteins may also be removed via the ER-associated degradative pathway (Bonifacino and Weissman, 1998). UPR-mediated alleviation of ER stress may allow the cell to survive, whereas prolonged ER stress can result in apoptosis (Gow and Sharma, 2003).

High-dose fluoride can trigger ER stress, which compromises ameloblast function during enamel development. Fluoride decreases KLK4 and TGF-β1 transcript and protein levels that are necessary for enamel formation (Suzuki et al., 2014a).

Chemical chaperones are small molecules and can eliminate aggregation and/or accumulation of misfolded proteins (Zhao et al., 2007) to cope with the ER stress. One such molecule is sodium 4-phenylbutyrate (4PBA). As a chemical chaperone, 4PBA helps with the correct folding of proteins to reduce ER stress (Kolb et al., 2015). 4PBA is a known inhibitor of histone deacetylase (HDAC) that could also affect gene expression (Daosukho et al., 2007). 4PBA is FDA-approved and is licensed for the treatment of urea cycle disorders (Iannitti and Palmieri, 2011), sickle cell disease (Odievre et al., 2007), and thalassemia (Collins et al., 1995). Amelogenesis imperfecta (AI) is an inherited disorder of enamel development with an incidence as high as 1 in 700 live births (Backman and Holm, 1986). A recent study demonstrated that AI pathogenesis is associated with ameloblast apoptosis induced by ER stress and 4PBA treatment rescued the AI enamel phenotype by inhibiting ER stress-mediated apoptosis in rodent model (Brookes et al., 2014).

To study dental fluorosis, rodent models have been employed because rodent incisors erupt continuously and every stage of enamel development is present along the length of the rodent incisor.

Here we assessed whether 4PBA protects against fluoride-mediated gene repression, apoptosis, and mitochondrial damage *in vitro*, and we analyzed 4PBA efficacy in the mouse dental fluorosis model.

## Materials and methods

### Reagents

Sodium fluoride (NaF) Cat. S299-100 was obtained from Fisher Scientific, (Pittsburgh, PA). 4-phenylbutyric acid sodium salt (4PBA) was purchased from Scandinavian Formulas, Cat. 1716-12-7 (Sellerville, PA).

### Animals

C57BL/6 mice (6-week-old) were purchased from Charles River Laboratories (Wilmington, MA). Mice (N = 5/group) were provided water containing 0, 50, 100 ppm fluoride as NaF *ad libitum* for 6 weeks. Animals were fed with either fluoride-free control-chow (F1515, rodent standard diet, AIN-76A, Bio-Sev, Frenchtown, NJ) or 4PBA-chow (same as control-chow containing 7 g/kg 4PBA, rodent custom diet, Bio-Sev). Mice were kept on these different chows beginning 1 week prior to fluoride water treatment until fluoride treatment termination. After fluoride treatment for 6 weeks, animals were euthanized and incisors were extracted for quantitative fluorescence (QF) analysis, Vickers microhardness measurments and measurement of fluoride concentration in bone, serum, and urine. All animals were treated humanely and all handling procedures were approved by the Institutional Animal Care Use Committee (IACUC) at The Forsyth Institute. The Forsyth Institute is accredited by the Association for Assessment and Accreditation of Laboratory Animal Care International (AAALAC) and follows the Guide for the Care and Use of Laboratory Animals (NRC1996). Note that the first and senior authors were employed by The Forsyth Institute through October 2015 when the animal experiments were completed.

### Cell culture

Mouse ameloblast-lineage cell line (ALC) Cells (Nakata et al., 2003) were grown in Dulbecco's modified Eagle's medium supplemented with 10% fetal bovine serum, 4.5 g/l of D-glucose, 4 mM L-glutamine, and 110 mg/l of sodium pyruvate (Invitrogen, Carlsbad, CA, USA) without antibiotics. Cells were treated with or without NaF (1–5 mM) in the presence or absence of 4PBA as indicated. 4PBA was present throughout the fluoride exposure. NaF 5 mM is corresponding to F^−^ 95 ppm.

### Real-time quantitative PCR (qPCR) analysis

Total RNA was extracted from cells using Direct-zol RNA Mini Prep (Zymo Research Corp., Irvine, CA).

Total RNA was reverse-transcribed into cDNA using a Transcriptor First Strand cDNA Synthesis Kit (Roche Diagnostics, Minneapolis, MN). The cDNA was subjected to qPCR amplification on a LightCycler 480 Real Time PCR System (Roche Diagnostics). The relative expression of target genes was determined by the 2^−ΔΔCT^ method (Pfaffl, 2001). The internal reference control gene was *B2m*. Primers (Invitrogen) and their sequences were;

*Bcl2* (Gene ID 12043),

forward: 5′-TCAGGCTGGAAGGAGAAGATG-3′ reverse: 5′-TGTCACAGAGGGGCTACGAGT-3′,

*Bax* (Gene ID 12028),

forward: 5′-AGCTGCCACCCGGAAGAAGACCT-3′ reverse: 5′-CCGGCGAATTGGAGATGAACTG-3′

*Tgf*-β*1* (Gene ID 21803),

forward: 5′-AGGACCTGGGTTGGAAGTGGAT-3′ reverse: 5′-AAGCGCCCGGGTTGTGTT-3′

*B2m* (Gene ID 12010),

forward: 5′- GGTCTTTCTGGTGCTTGTCTC -3′ reverse: 5′- CGTAGCAGTTCAGTATGTTCG G -3′.

Three biological replicates were analyzed. Data were presented as the mean ± standard deviation (SD).

### Western blot analysis

Western blots were performed as described previously (Suzuki et al., 2015). Briefly, mitochondrial fractions and cytosolic fractions were isolated using a mitochondria isolation kit for cultured cells (Thermo Scientific, Rockford, IL). Equal amounts of protein per lane (5–20 μg) were loaded onto Mini-Protean® TGX™ gels (Biorad, Hercules, CA), transferred to Trans-Blot Turbo Transfer nitrocellulose membranes (Biorad) and probed with primary antibodies. Primary antibodies included: rabbit anti-cytochrome-c, rabbit anti-VDAC1/Porin (Abcam, Inc., Cambridge, MA), and rabbit anti-β-actin (Cell Signaling Technology, Danvers, MA). The secondary antibody was HRP-conjugated goat anti-rabbit IgG (Biorad). Enhanced chemiluminescence was performed with SuperSignal West Pico (Thermo Scientific). Signal was detected by myECL imager (Thermo Scientific) and band density was quantified using myimageAnalysis™ Software (Thermo Scientific).

### Photographs of mouse incisors

After fluoride water treatment with control-chow or 4PBA-chow for 6 weeks, animals were euthanized. Heads were cleaned of fur and skin. Photographs of the maxillary and mandibular incisors were taken using a Nikon SMZ745T microscope and Leica DFC400 digital camera under standard white balance and lighting conditions.

### QF assay and measurement of fluoride concentration

Quantitative fluorescence (QF) has been used to evaluate the severity of fluorosis in mice (Everett et al., 2002). Mandibular incisors were dissected as pairs and subjected to QF using a Nikon epifluorescence micro camera equipped with a Chroma Gold 11006v2 set cube (exciter D360/40x, dichroic 400DCLP, and emitter E515LPv2). Fluorescence images of teeth were converted to grayscale values and intensities were analyzed using Image J software (http://imagej.nih.gov/ij/). Samples of mouse chow, serum, urine and bone (femurs) were assessed for fluoride concentration as previously described (Sharma et al., 2011).

### Vickers microhardness testing of mouse incisor enamel

Erupted portions of mandibular and maxillary incisors from mice were washed and dehydrated with graded alcohol and acetone. Incisors were embedded sagittally in hard-formulation epoxy embedding medium (EpoFix, EMS, Hatfield, PA) and samples were ground and polished to 0.25 μm with diamond suspensions (EMS) as previously described (Shin et al., 2014). The polished samples were tested for enamel microhardness on an M 400 HI testing machine (Leco, St. Joseph, Michigan). Testing was performed with a load of 25 g for 5 s with a Vickers tip. Twelve indentations per sample were performed on five teeth per group and averaged. Data are presented as the mean ± standard deviation (SD).

### Statistical analysis

Quantitative analysis between two groups was performed by Student's *t*-test. Multiple group comparison was performed by one-way analysis of variance with Fisher's protected least significant difference post hoc test. Significance was assessed at *P* < 0.05.

## Results

### 4PBA reversed fluoride-induced *Tgf-β1* suppression in ALC cells

**Figure 1:**
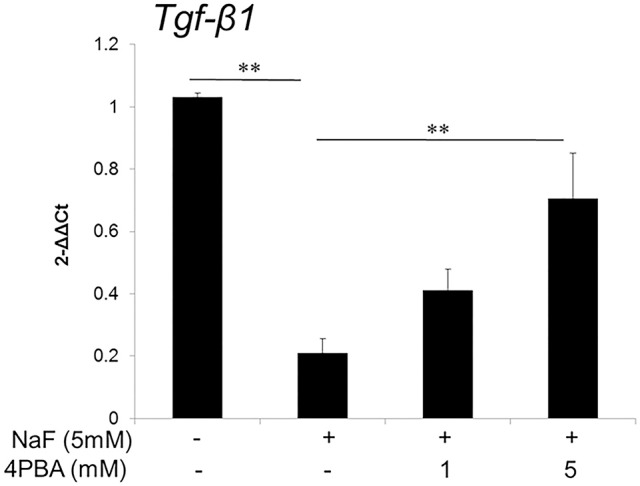
**4PBA reversed fluoride-induced *Tgf*-β*1* suppression in ALC cells**. ALC cells were treated with 4PBA (0–5 mM) for 1 h followed by the addition of 5 mM (95 ppm) fluoride for 24 h. *Tgf*-β*1* mRNA was quantified by qPCR. Fluoride decreased *Tgf*-β*1* expression and 4PBA treatment significantly reversed the suppression of *Tgf*-β*1* expression. Three biological replicates were analyzed. Data are presented as the mean ± standard deviation (SD). Results were analyzed by one-way analysis of variance with Fisher's protected least significant difference *post hoc* test. ^**^P < 0.01.

Previously we reported fluoride treatment decreased *Tgf*-β*1* transcript and protein levels, which is associated with enamel malformation (Suzuki et al., 2014a). Here we asked if 4PBA prevents fluoride-induced *Tgf*-β*1* repression. ALC cells were treated with 4PBA for 1 h followed by addition of 5 mM (95 ppm) fluoride treatment for 24 h. *Tgf*-β*1* mRNA expression was decreased by fluoride treatment, but this was reversed by 4PBA treatment in a dose-dependent manner (*P* < 0.01; Figure 1).

### 4PBA increased anti-apoptotic gene expression ratio (*Blc2/Bax*) in ALC cells

**Figure 2:**
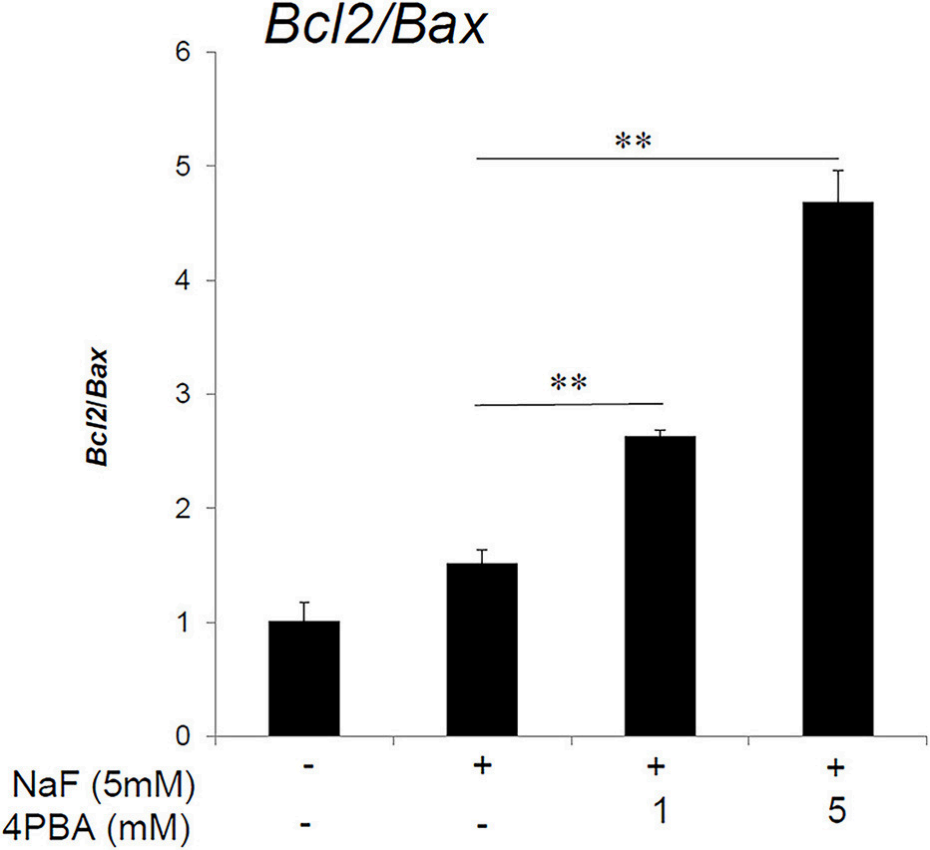
**Anti-apoptotic *Blc2/Bax* expression ratio increased by 4PBA treatment of ALC cells**. ALC cells were treated with 4PBA (0–5 mM) for 1 h followed by the addition of 5 mM (95 ppm) fluoride for 24 h. *Blc2* and *Bax* mRNA were quantified by qPCR. The ratio of *Blc2/Bax* was significantly increased with NaF/4PBA treatment compared to NaF treatment alone. Three biological replicates were analyzed. Data are presented as the mean ± standard deviation (SD). Results were analyzed by one-way analysis of variance with Fisher's protected least significant difference *post hoc* test. ^**^P < 0.01.

High-dose fluoride induces apoptosis in ameloblast-derived cell line (LS8) cells (Suzuki and Bartlett, 2014) and in rodent ameloblasts (Kubota et al., 2005). Anti-apoptotic Bcl-2 protein can repress apoptotic death programs, while pro-apoptotic Bax protein can accelerate cell death. The Bcl2/Bax ratio determines survival or death following an apoptotic stimulus (Oltvai et al., 1993). Next, we assessed the 4PBA effect on the *Bcl2*/*Bax* expression ratio in ALC cells. The qPCR results showed that fluoride treatment alone did not significantly alter the anti-apoptotic *Blc2/Bax* ratio compared to the non-fluoride-treated control, however 4PBA treatment significantly increased the *Blc2/Bax* ratio compared to fluoride alone (*P* < 0.01; Figure 2). Previously we demonstrated that fluoride treatment alone induces apoptosis with accompanying caspase-3 cleavage (Suzuki and Bartlett, 2014) and DNA fragmentation (Kubota et al., 2005). In the current study, contrary our expectation, fluoride treatment alone did not significantly alter the Blc2/Bax ratio. Since there are several apoptotic pathways and apoptotic factors besides Bcl2 and Bax, we interpret that Bcl2 and Bax may not be main factors in fluoride-induced pro-apoptotic pathways in ALC cells. However, 4PBA can counteract fluoride-induced apoptosis by increasing anti-apoptotic Bcl2/Bax ratio.

### Fluoride-induced cytochrome-c release was inhibited by 4PBA treatment in ALC cells

**Figure 3:**
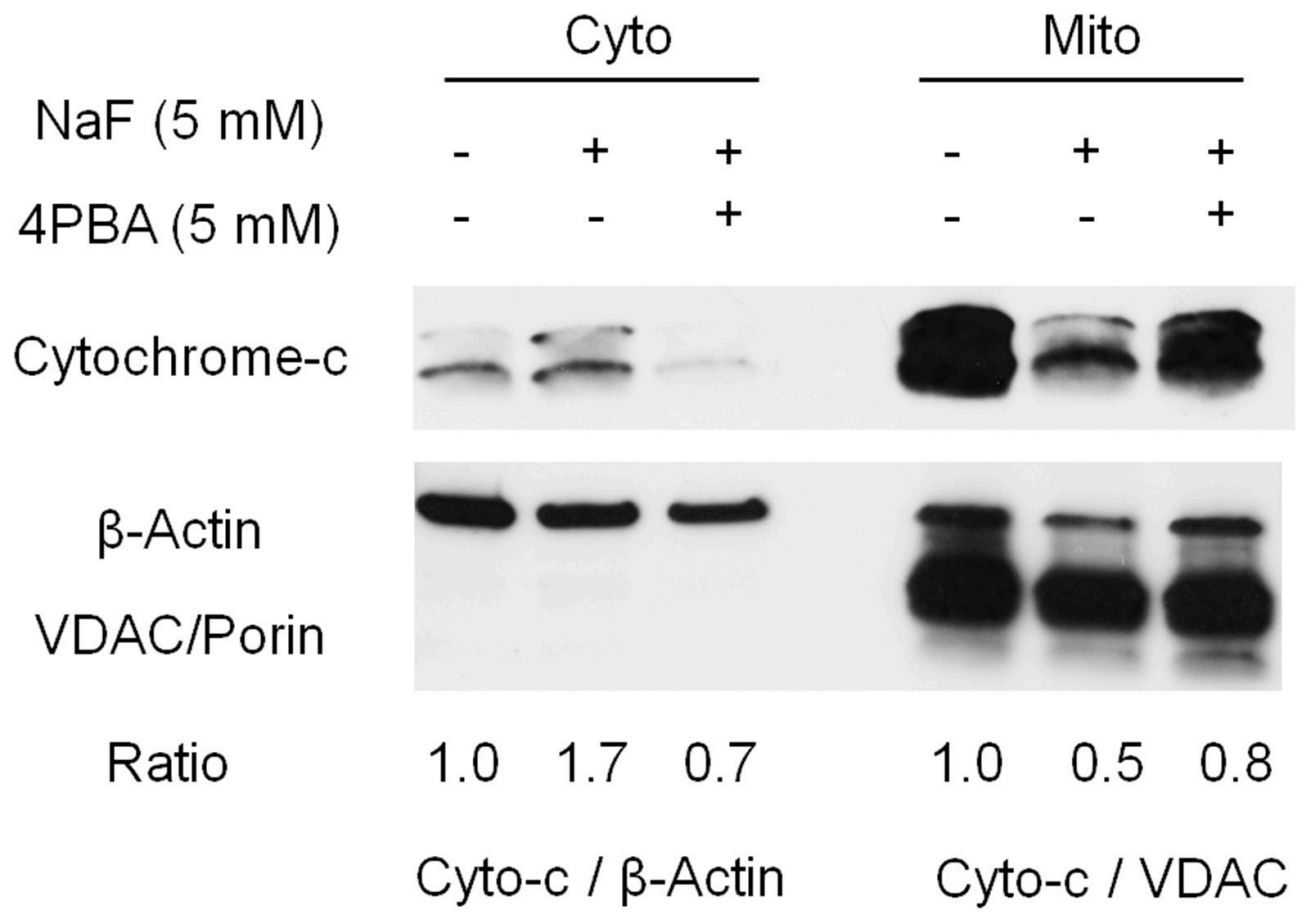
**Fluoride-induced cytochrome-c release was inhibited by 4PBA in ALC cells**. ALC cells were treated with 5 mM 4PBA for 1 h followed by the addition of 5 mM (95 ppm) of fluoride for 24 h. Cytochrome-c (12 kDa) in the cytosol (Cyto) and in the mitochondria (Mito) was detected by Western blots. Fluoride induced cytochrome-c release from Mito into Cyto, which was reversed by 4PBA pretreatment. β-Actin (42 kDa) and VDAC/Porin (31 kDa) were used as loading controls for Cyto and Mito respectively. Figure shows the representative result of three biological experiments. Numbers indicate cytochrome-c/β-actin expression ratio (for cytosol) or cytochrome-c/VDAC expression ratio (for mitochondria).

High-dose fluoride induces oxidative stress (Suzuki et al., 2014b) followed by mitochondrial damage (Suzuki et al., 2015). Here, we asked if 4PBA mitigates fluoride-induced cytochrome-c release in ALC cells. Cells were treated with 4PBA for 1 h followed by 5 mM (95 ppm) fluoride for 24 h. Western blot results showed that fluoride treatment alone increased cytochrome-c in the cytosol fraction (Cyto) and decreased it in the mitochondrial fraction (Mito). In contrast, 4PBA treatment prevented fluoride-induced cytochrome-c release into cytosol fraction (Figure 3). This result indicates that 4PBA protects ameloblasts from fluoride-induced mitochondrial damage.

### Effects of fluoride and 4PBA on rodent tissues

**Figure 4:**
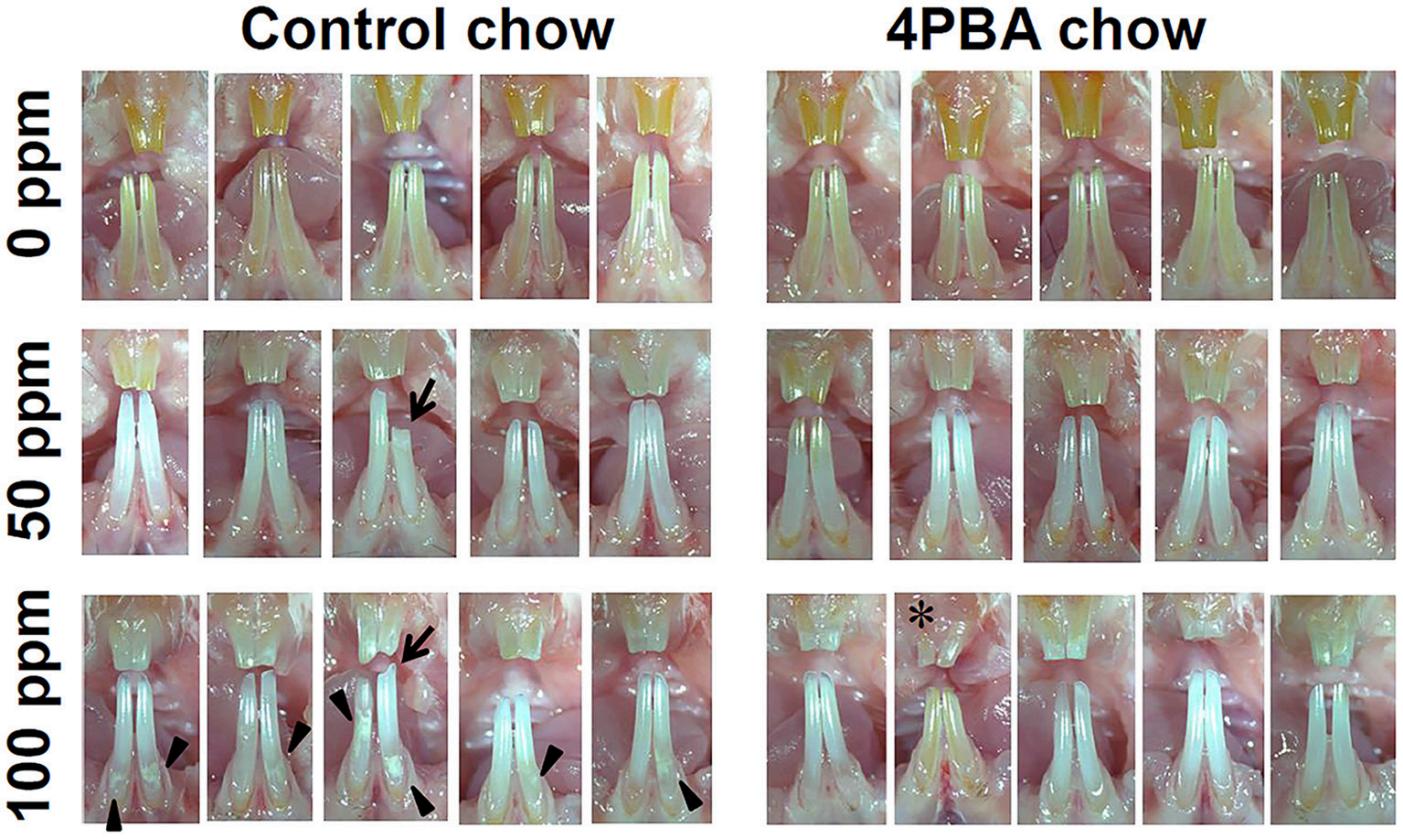
**Incisor phenotype of mice treated with fluoride with either control-chow or 4PBA-chow**. C57BL/6 mice (*N* = 5/group) were provided water *ad libitum* containing 0 (upper row), 50 (middle row) or 100 (bottom row) ppm fluoride and were fed for 6 weeks with either control-chow (left panels) or 4PBA-containing chow (7 g/kg) (right panels). After 6 weeks, animals were euthanized. Pictures are of five mouse incisors for each group.

**Figure 5:**
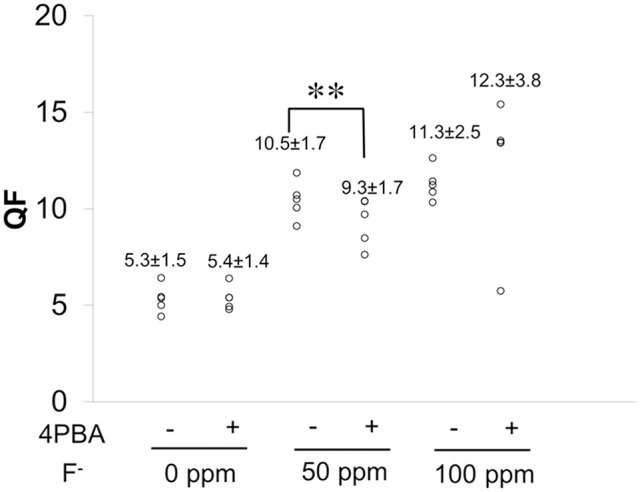
**QF analysis of enamel**. C57BL/6 mice (*N* = 5/group) were provided water *ad libitum* containing fluoride (0, 50, or 100 ppm) with either control-chow or 4 PBA-chow for 6 weeks. After 6weeks, fluorescence of mandibular incisors was quantified by QF assay. Data are presented as scatter plots from five mice in each group. Numbers indicate the mean ± standard deviation (SD). Results between control-chow and 4-PBA chow were analyzed by Student's *t*-test. *P* < 0.05 was considered significant. ^**^P < 0.01.

**Figure 6:**
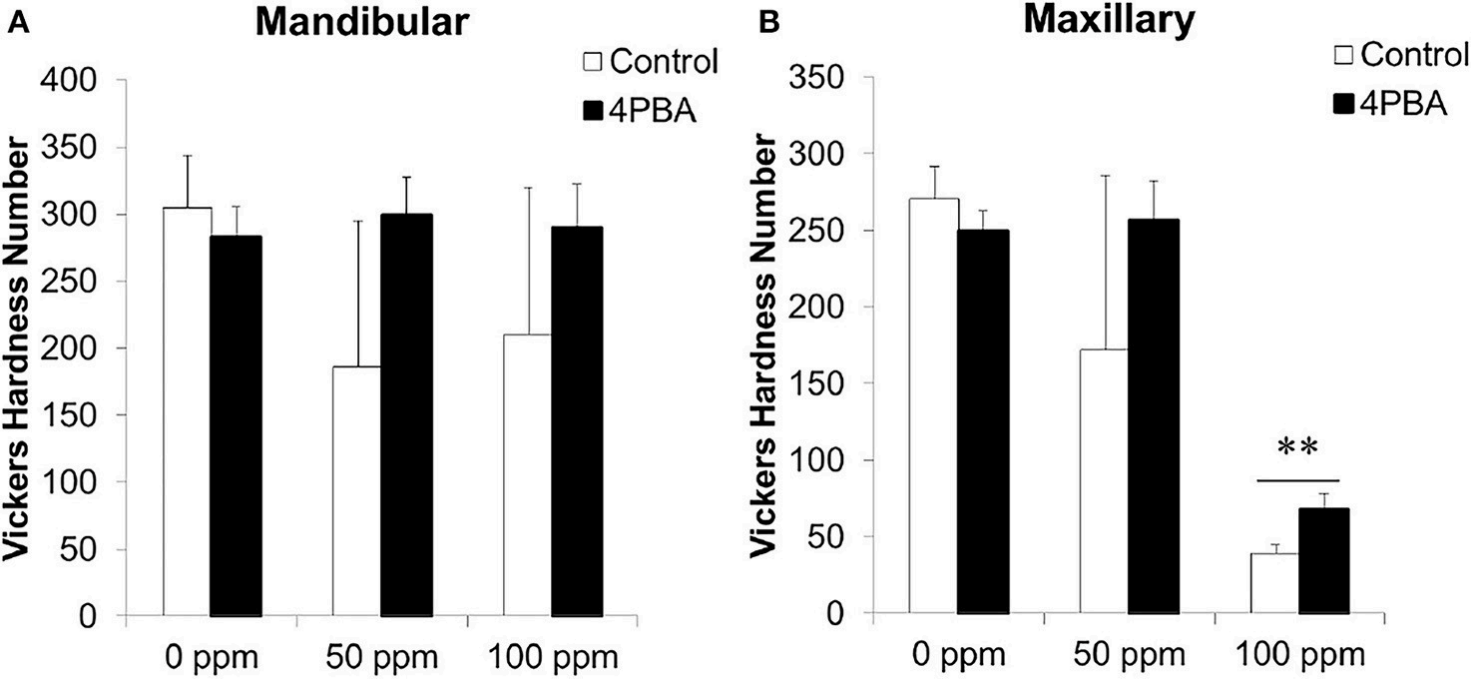
**Microhardness measurement on incisor longitudinal sections**. C57BL/6 mice (*N* = 5/group) were provided water *ad libitum* containing fluoride (0, 50, or 100 ppm) with either control-chow (open columns) or 4 PBA-chow (filled columns) for 6 weeks. After 6weeks, microhardness of mandibular **(A)** and maxillary **(B)** incisors was measured. Data are presented as the mean ± standard deviation (SD). Results between control-chow and 4PBA chow were analyzed by Student's *t*-test. *P* < 0.05 was considered significant.^**^P < 0.01.

**Figure 7:**
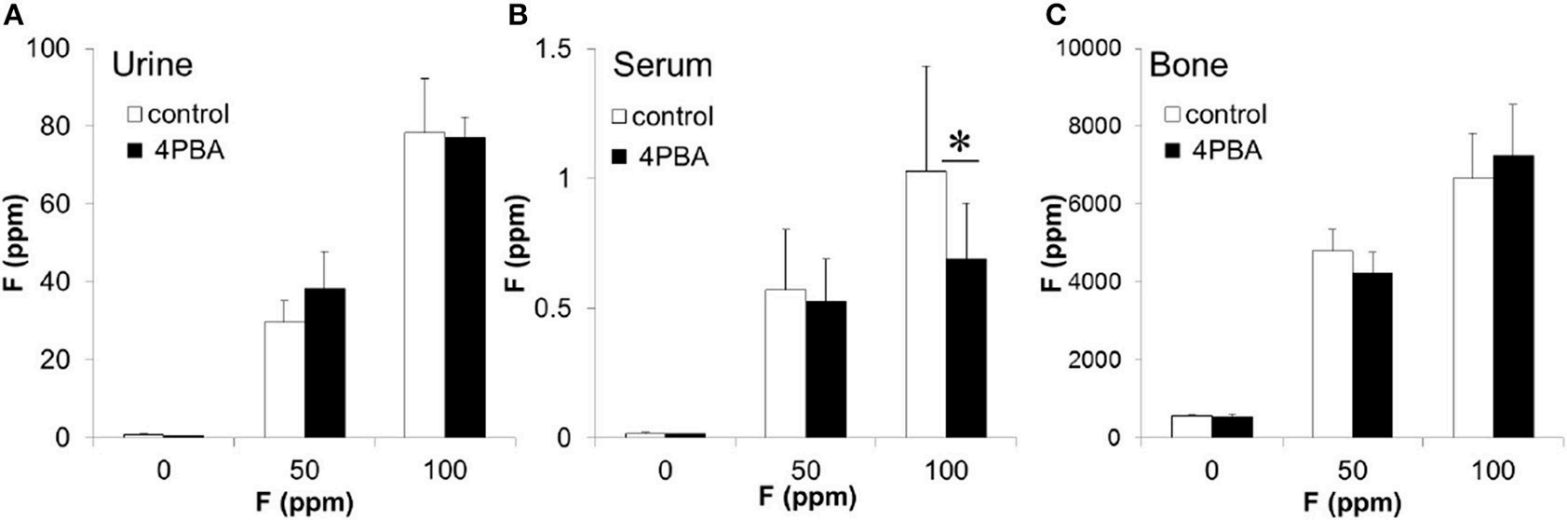
**The concentration of fluoride in mouse urine, serum and bone**. C57BL/6 mice (*N* = 5/group) were provided water *ad libitum* containing fluoride (0, 50, or 100 ppm) with either control-chow (open columns) or 4 PBA-chow (filled columns) for 6 weeks. After 6 weeks, fluoride concentrations (ppm) in urine **(A)**, serum **(B)** and bone **(C)** were measured. Data are presented as the mean ± standard deviation (SD). Results between control-chow and 4-PBA chow were analyzed by Student's *t*-test. *P* < 0.05 was considered significant. ^*^P < 0.05.

Next we evaluated the 4PBA efficacy in a rodent dental fluorosis model. After fluoride treatment, animals were euthanized and incisor phenotype (Figure 4), fluorosis level (Figure 5), enamel microhardness (Figure 6), and fluoride concentrations in serum, urine and bone (Figure 7) were assessed.

### Incisor phenotype of mice treated with fluoride water with either control-chow or 4PBA-chow

Figure 4 shows five mouse incisors for each treatment group. Left panels show control-chow groups and right panels show 4PBA-chow groups. Among control-chow groups, compared to the 0 ppm fluoride group (upper row), tooth color was changed to chalky white opaque in both 50 ppm (middle) and 100 ppm (bottom) fluoride groups. Attrition (indicated by arrow) was observed in 50 ppm and 100 ppm groups and white spots (indicated by arrow head) were detected in the 100 ppm group. Among 4PBA-chow groups (right panels), fluoride treatment (50 ppm and 100 ppm) changed tooth color to chalky white opaque, however attrition and white spots were not seen in 4PBA-chow groups. In addition, in the 100 ppm fluoride/4PBA chow group (bottom in right panel), there was a mouse with pale creamy colored teeth (indicated by ^*^) similar to teeth in the 0 ppm group.

### Fluorosis level quantified by QF assay

Figure 5 shows fluorosis level as measured by QF assay. Data are presented as scatter plots from five mice in each group. Numbers indicate the mean ± standard deviation (SD). Fluoride treatment increased QF in a dose-dependent manner in both control-chow groups (−) and in 4PBA-chow groups (+). Between control-chow (−) and 4PBA-chow (+), the 4PBA-chow significantly (^**^P < 0.01) decreased QF in the 50 ppm fluoride treatment but not in the 100 ppm group.

### Measurement of enamel hardness

Previously we demonstrated that fluorosed mouse incisor enamel is significantly softer than normal (Bartlett et al., 2004; Tye et al., 2009). Microhardness of fluoride-treated enamel significantly decreased as compared with control enamel (Sharma et al., 2011). Here we assessed the 4PBA effect on enamel hardness as a function of fluoride treatment. Figure 6 shows Vickers microhardness values from mouse mandibular (A) and maxillary (B) incisors. Each bar represents hardness measurements for incisors from 5 mice in each group. Between control-chow (open columns) and 4PBA-chow (filled columns), the 4PBA-chow significantly (*P* < 0.01) increased enamel hardness compared to control-chow in only maxillary incisors treated with 100 ppm fluoride (Figure 6B).

### Fluoride concentrations in mouse urine, serum, and bone

After fluoride treatment, fluoride concentrations (ppm) in urine (A), serum (B), and bone (C) were measured (Figure 7). Compared to control-chow (open columns), 4PBA-chow (filled columns) had no effect on the quantity of fluoride that accumulated in the urine or bone (Figures 7A,C). However, 4PBA-chow significantly decreased fluoride concentration (*P* < 0.05) in serum exposed to 100 ppm fluoride treatment (Figure 7B). Trace amounts of fluoride were found in control-chow (1.16 ppm), 4PBA-chow (1.36 ppm) and control water (0.01 ppm). For fluoride at 50 ppm and at 100 ppm in water, we directly measured concentrations of 51.69 and 105.26 ppm respectively.

## Discussion

In the present study, we hypothesized that 4PBA is an effective treatment for dental fluorosis and tested if 4PBA ameliorates fluoride toxicity in ALC cells and in mouse dental fluorosis. Previously we demonstrated that high-dose fluoride induces ER stress and oxidative stress in ameloblasts that results in *Klk4* and *Tgf*-β*1* repression (Sharma et al., 2010; Suzuki et al., 2014a), mitochondrial damage (cytochrome-c release), DNA damage and apoptosis (Suzuki et al., 2015). In the present study, we demonstrated that 4PBA pretreatment reversed fluoride-induced *Tgf*-β*1* repression (Figure 1), increased the anti-apoptotic *Bcl2/Bax* expression ratio in ALC cells (Figure 2) and inhibited cytochrome-c release (Figure 3). However, the mechanism of how 4PBA alleviated fluoride toxicity in ALC cells remained to be elucidated. Since 4PBA is a chemical chaperone and helps with the correct folding of proteins to reduce ER stress (Kolb et al., 2015), 4PBA could attenuate fluoride-induced ER stress to alleviate gene repression, apoptosis and mitochondrial damage. On the other hand, 4PBA is a known inhibitor of histone deacetylase (HDAC) (Daosukho et al., 2007). 4PBA is a short chain fatty acid derivative that inhibits class I and class IIa HDACs, but not IIb HDACs (Bolden et al., 2006; Chuang et al., 2009). HDAC inhibitors increase the acetylation of histone and non-histone proteins to activate transcription, enhance gene expression, and modify the function of target proteins. HDAC inhibitors provide protection against not only ER stress but also oxidative stress to promote survival over cell stress (Fessler et al., 2013). Although it is largely unknown if and how 4PBA targets class I and class IIa HDACs during the pathology of dental fluorosis, the results *in vitro* suggest that 4PBA may ameliorate fluoride-induced *Tgf*-β*1* repression, apoptosis, and mitochondrial damage in ALC cells.

Next we asked if 4PBA mitigates dental fluorosis in a rodent model. Figure 4 shows the phenotype of mouse incisors treated with fluoride and fed with either control-chow or 4PBA-chow. Contrary to our expectation, fluoride treatment changed tooth color to a chalky white opaque color in both control-chow and 4PBA-chow groups. However, attrition and white spots were observed only in the fluoride/control-chow groups but not in the fluoride/4PBA-chow groups. Moreover, in the 100 ppm fluoride/4PBA-chow group, we observed a mouse with incisors of a color similar to non-fluoride treated mice. Statistical analysis shows that 4PBA-chow significantly decreased fluorosis levels in the 50 ppm fluoride treatment group (Figure 5) and reversed microhardness in maxillary incisors treated with 100 ppm fluoride (Figure 6B) and also decreased serum fluoride concentrations in the 100 ppm fluoride treatment group (Figure 7B). Taken together, 4PBA might act to avert fluoride toxicity, but results were not consistent across *in vivo* analyses. In contrast to *in vitro* results, with few exceptions, 4PBA did not ameliorate dental fluorosis in our mouse model. Perhaps, not enough intact 4PBA reaches the rodent ameloblasts to reverse the effects of fluoride toxicity. In addition, careful consideration should be given to the HDAC inhibitor function of 4PBA. Recently, we demonstrated that fluoride activates SIRT1 in ameloblasts as an adaptive response and pharmacological SIRT1 activation protects ameloblasts from fluoride-induced cell stress (Suzuki and Bartlett, 2014; Suzuki et al., 2015). SIRT1 is a highly conserved NAD^+^-dependent class III HDAC. By deacetylating target substrates, SIRT1 promotes cell survival by modulating cellular processes involved in stress adaptation (Michan and Sinclair, 2007). Even though it has not been reported if 4PBA can affect SIRT1 (class III HDAC), it seems prudent to assess the effect of 4PBA on SIRT1 function in dental fluorosis.

In conclusion, we show that 4PBA mitigated fluoride toxicity *in vitro*, while in general, a diet rich in 4PBA did not attenuate dental fluorosis in rodents. Further studies will be required to optimize protocols for 4PBA administration *in vivo* in order to evaluate the effect of 4PBA on dental fluorosis.

## Ethics statement

This study was carried out in accordance with the recommendations of Institutional Animal Care Use Committee (IACUC) at the Forsyth Institute. The Forsyth Institute is accredited by the Association for Assessment and Accreditation of Laboratory Animal Care International (AAALAC) and follows the Guide for the Care and Use of Laboratory Animals (NRC1996). The protocol was approved by the IACUC at the Forsyth Institute. Note that the first and senior authors were employed by The Forsyth Institute through October 2015 when the animal experiments were completed.

## Author contributions

MS and JB designed experiments. MS, EE, and GW performed the experiments. MS prepared the figures and wrote the main manuscript text. JB reviewed and modified the manuscript text. All authors reviewed the manuscript.

## Funding

Research reported in this publication was supported by the National Institute of Dental and Craniofacial Research of the National Institutes of Health under Award R01DE018106 (JB).

### Conflict of interest statement

The authors declare that the research was conducted in the absence of any commercial or financial relationships that could be construed as a potential conflict of interest.
